# Age-Specific Concentrations and Seroprevalence of Antibodies Against *Salmonella* Enteritidis (O:9) and *Salmonella* Typhimurium (O:4,5) Across 3 Sites in Kenya

**DOI:** 10.1093/infdis/jiag114

**Published:** 2026-03-04

**Authors:** Esther M Muthumbi, Sean C Elias, Alfred Mwanzu, Agnes Mutiso, Perpetual Wanjiku, Cecilia Mbae, Godfrey Bigogo, Jennifer R Verani, Stefan Flasche, Samuel Kariuki, Calman A MacLennan, J Anthony G Scott

**Affiliations:** Department of Epidemiology and Demography, Kenya Medical Research Institute–Centre for Geographic Medicine Research, Coast, Kilifi, Kenya; Department of Infectious Disease Epidemiology, London School of Hygiene and Tropical Medicine, United Kingdom; Department of Population Health, Aga Khan University, Nairobi, Kenya; Jenner Institute, Nuffield Department of Medicine, University of Oxford, United Kingdom; Department of Epidemiology and Demography, Kenya Medical Research Institute–Centre for Geographic Medicine Research, Coast, Kilifi, Kenya; Department of Epidemiology and Demography, Kenya Medical Research Institute–Centre for Geographic Medicine Research, Coast, Kilifi, Kenya; Department of Epidemiology and Demography, Kenya Medical Research Institute–Centre for Geographic Medicine Research, Coast, Kilifi, Kenya; Kenya Medical Research Institute–Centre for Microbiology Research, Nairobi; Kenya Medical Research Institute–Centre for Global Health Research, Kisumu; Division of Global Health Protection, US Centers for Disease Control and Prevention, Nairobi, Kenya; Department of Infectious Disease Epidemiology, London School of Hygiene and Tropical Medicine, United Kingdom; Centre for Global Health at Charite Universitaetsmedizin Berlin, Germany; Kenya Medical Research Institute–Centre for Microbiology Research, Nairobi; Jenner Institute, Nuffield Department of Medicine, University of Oxford, United Kingdom; Department of Immunology and Immunotherapy, University of Birmingham, United Kingdom; Department of Epidemiology and Demography, Kenya Medical Research Institute–Centre for Geographic Medicine Research, Coast, Kilifi, Kenya; Department of Infectious Disease Epidemiology, London School of Hygiene and Tropical Medicine, United Kingdom

**Keywords:** Africa, antibodies, nontyphoidal, *Salmonella*, vaccines

## Abstract

**Background:**

Seroprevalence studies can indicate the age distribution of first infection with nontyphoidal *Salmonella* and estimate the rate of infection by age.

**Method:**

We collected 1254 paired samples of serum and stool from healthy children and adults in Kilifi, Nairobi, and Siaya counties in Kenya, areas of low, medium, and high incidence of invasive nontyphoidal *Salmonella* disease, respectively. We quantified the age- and site-specific geometric mean concentrations (GMCs) of IgG and IgA antibodies against *S* Enteritidis O-antigen (O:9, serogroup D) and *S* Typhimurium O-antigen (O:4,5, serogroup B) using an in-house standardized enzyme-linked immunosorbent assay. Serum concentrations were estimated against a previously calibrated reference serum.

**Results:**

Maternally derived O:9 IgG and O:4,5 IgG antibodies were detectable in 100% of neonates, and the GMC decreased by 40% (95% CI, 25%–52%) per month in the first 6 months of life. GMCs of IgA were low in neonates. After age 6 months, the O:9 and O:4,5 IgG and IgA GMCs increased sharply with age across all sites, reaching a plateau in early adulthood. The rate of increase in IgG GMCs by age was highest for O:9 in Nairobi and for O:4,5 in Kilifi. Mixture modeling defined a threshold of 14.1 antibody units for O:9 IgG and 28.2 antibody units for O:4,5 IgG. Seroprevalence also increased by age. The GMCs of O:4,5 IgG were 2 times higher for carriers of serogroup B *Salmonella* than noncarriers.

**Discussion:**

Maternal antibodies to nontyphoidal *Salmonella* decay rapidly from 0 to 5 months, after which incident infection with both serogroups occurs. To be effective, control efforts should be implemented before primary infection.

## Teaser

Serologic evidence shows age-related increases in IgG against nontyphoidal *Salmonella* O-antigens, with a postinfancy nadir and subsequent rise. Patterns indicate cumulative exposure and early vulnerability, informing optimal vaccine targeting and establishing serology as a tool to monitor transmission.

Invasive nontyphoidal *Salmonella* (NTS) disease is a food-borne illness that causes significant morbidity and mortality globally, estimated as 535 000 cases and 77 500 deaths in 2017 [1]. The burden is highest in sub-Saharan Africa and in children <5 years old, and >80% is caused by 2 serovars: *S* Typhimurium and *S* Enteritidis [1, 2]. Ingestion of NTS bacteria can lead to transient mucosal infection and clearance, asymptomatic colonization, enterocolitis, or invasive disease [3, 4]. Hospital-based surveillance captures symptomatic cases, such as those with diarrhea and those with bacteremia, while community-based cross-sectional studies among healthy individuals estimate the prevalence of asymptomatic carriage [5, 6]. Culture-based estimates of NTS burden are limited by the low sensitivity of culture for isolation of NTS. Additionally, hospital-based estimates are limited by access to diagnostic facilities, leading to underestimation of the burden of NTS. Quantification of IgG and IgA antibodies by age reflects the cumulative incidence of infection in the community [7, 8]. Analysis of age-related serologic profiles for respiratory syncytial virus, for example, has been used to estimate the rate of infection by age and guide the optimal age to target interventions, particularly vaccination [9]. In addition, for vaccines that might be designed to interrupt human-to-human transmission of NTS [10, 11], understanding the relationship between antibodies and gut clearance is also critical.

Currently no vaccine against NTS has been licensed, although there are several candidates in different stages of development [9, 10]. All target the serovars responsible for the majority of invasive NTS cases—namely, *S* Typhimurium (serogroup B, O:4,5) and *S* Enteritidis (serogroup D_1_, O:9)—either in bivalent formulations or in multivalent formulations including typhoidal serovars. These vaccines aim to prevent disease in infants, in whom incidence is highest [12]. However, maternal antibodies may reduce the efficacy of infant vaccination [13]. Assessment of the decay of maternally derived IgG antibodies in infants against the rate of acquisition of IgA antibodies in infants can identify the optimal window for vaccination with a future NTS vaccine [14].

Serologic approaches provide an important means of characterizing population exposure to NTS. However, interpretation of anti-NTS antibody data is challenging. There are no standardized assays for the quantification of anti-NTS antibodies [15] and no commonly agreed threshold for defining seropositivity or protection against disease. In endemic settings, identifying truly unexposed individuals (appropriate negative controls) is particularly difficult, as exposure to NTS occurs early in life, resulting in widespread background antibody responses. In addition, antibodies targeting NTS O-antigens may reflect cumulative exposure to multiple serotypes within a serogroup and cross-reactive responses to other gram-negative organisms, further complicating interpretation. Robust approaches that integrate assay design, control selection, and analytical strategies are therefore required to quantify infection dynamics, compare transmission intensity across settings, and inform the optimal timing and targeting of future NTS control strategies, including vaccination.

The aim of this study was to estimate the age-specific geometric mean concentrations and seroprevalence of anti-NTS antibodies across 3 sites in Kenya to inform future vaccine deployment strategies.

## METHODS

We selected 3 sites in Kenya—Kilifi, Nairobi, and Siaya—where the incidence of invasive NTS was 36 to 88 [12], 255 to 998, and 501 to 3914 per 10^5^ person-years of observation [16], respectively. A randomly selected age-stratified sample of healthy children was recruited into a community-based cross-sectional study of the prevalence of fecal carriage of NTS (n = 1497) [5]. This involved collection of stool samples for isolation of NTS, anthropometry measures, and a venous blood sample for testing of anti-NTS antibodies, spot testing for malaria parasites and hemoglobin concentrations. The samples were transported to the laboratory in cool boxes at 4 °C, where the serum was separated, aliquoted, and stored at −80 °C.

### Laboratory Methods

We used an indirect enzyme-linked immunosorbent assay (ELISA) for detection of anti-O:4,5 antibodies representing *S* Typhimurium and anti-O:9 antibodies representing *S* Enteritidis [17]. Plates were coated with purified O-antigens (CVD1925 and CVD1943) [18]. Nunc Maxisorb 96-well plates were coated with 5-μg/mL O-antigen in coating buffer (5.3-g Na_2_CO_3_ + 4.2-g NaHCO_3_ + 1-L dH_2_0, pH 9.6) and incubated overnight at +4 °C. The plates were washed 5 times with phosphate-buffered saline (PBS) containing 0.05% Tween 20 (PBS-Tween), blocked with casein (37528; Thermo Scientific) for 1 hour, and washed again 5 times with PBS-Tween and ×1 with PBS. Test sera were initially diluted at 1:50 and plated in triplicates. Where higher dilutions were required, 1:500 or 1:5000 dilutions of the sera were used.

An in-house human control serum containing antibodies to the 2 antigens was used as reference sera for the standard curve [17, 19]. These were plated in duplicate following a series of eleven 2-fold dilutions starting at 1:10 (or 1:20 for the O:4,5 IgG assay). Controls were included on each plate as follows: a negative control, consisting of casein dilution buffer added to 2 “blank” wells; a positive control, consisting of pooled serum diluted in casein in 2 wells from Malawian adults who were positive for invasive NTS (culture-confirmed NTS bacteremia); an internal control, consisting of the reference sera diluted at 1:160 for IgA assays and 1:320 for IgG assays plated in 4 wells.

The plates were then incubated for 2 hours at room temperature and subsequently washed with PBS-Tween ×5 and PBS ×1. A corresponding secondary antibody—either the anti-human IgA (α-chain specific) peroxidase antibody produced in goat (A0295; Sigma) or the anti-human IgG (γ-chain specific) peroxidase antibody produced in goat (A6029; Sigma)—was added to the plates, which were then incubated at room temperature for 1 hour, followed by a washing step with PBS-Tween ×5. The plates were developed with TMB Substrate (ab171522; Abcam) for the color reaction, which was controlled by addition of the stop solution (450-nm TMB Stop Solution, ab171529; Abcam). The development time was calibrated to generate an optical density (OD) of 1 in the internal control.

Once the stop solution was added, the plates were read within 30 minutes by an ELISA reader operating Gen5 Software (version 2.0). Bound antibodies were quantified by measuring the absorbance at 450 nm, minus the OD of the negative control (blank wells), and interpolating the values relative to the reference sera based on a 4-parameter logistic regression curve as ELISA antibody units (AUs). One AU is equal to the reciprocal of the dilution of the standard serum, giving an OD = 1. Repeat testing of the samples was done if the coefficient of variation between the triplicates was >20%. Full plate repeats were done if the fit of the standard curve was unsatisfactory (*R*^2^ < 0.994), if the background reaction was high (negative control >0.15 OD), or if the reference serum and positive controls were not within the defined range. Additional dilutions of the samples were recommended if the OD of the sample exceeded the OD of the first dilution point of the standard curve. Those below the bottom end of the reference curve were considered negative.

### Statistical Analysis

The AUs were log transformed (base 10). For log transformation, zero values were allocated a value that was half of the assay's lower limit of quantitation. Geometric mean concentrations were estimated by the age strata used in sampling (0–11 months, 12–59 months, 5–14 years, 15–54 years, and ≥55 years), by site (Kilifi, Nairobi, Siaya), and by fecal NTS carriage status. Comparisons among groups were performed by analysis of variance with Tukey pairwise comparison tests. We used the reverse cumulative distribution curves to visualize the distribution of antibody concentrations by age group.

A piecewise regression model, with a single inflection point at age *p*, was used to assess the rate of change of antibody levels with age (*x*). It fit 2 linear regressions to data, one where *x* < *p* and one where *x* ≥ *p*, per the *nl* command in Stata. It estimates the age at inflection/breakpoint *p*, the gradient in the age band where *x* < *p*, and the gradient in the age band where *x* ≥ *p*. To account for maternal antibodies, we excluded the period <6 months (*m* > *x* > *p*) [14].

Linear regression on log antibody concentrations was used to assess the mean rate of decay, *d*, of maternal antibodies, according to the following formula [20]:


$$(x_{a})=(x_{o}).e^{-da},$$

where (*x_a_*) refers to the mean antibody concentration at age *a* while (*x_o_*) refers to the mean antibody concentration at birth. These models were fit separately for each site.

Given the absence of standardized serologic thresholds and the high background exposure in endemic populations, we used finite mixture models to estimate seroprevalence [21, 22]. A 2-component Gaussian mixture model was constructed to separate the data into 2 latent classes, assumed to be a seronegative class (lower antibody component) and a seropositive class (higher antibody component). This approach uses the data to reconstruct the subclasses assuming that their distributions are normal and different from each other, deriving a mean and standard deviation for each subclass via the *fmm* package in Stata (version 15) [23]. By assuming that these lower and higher antibody components represent the best available approximation of seronegative and seropositive populations, respectively, sensitivity for a given cutoff was defined as the probability that values drawn from the higher antibody distribution exceed the cutoff, and specificity was defined as the probability that values drawn from the lower antibody distribution fall below the cutoff. These probabilities were used to construct receiver operating characteristic curves and identify optimal cutoffs via the Youden index [24] and to estimate the sensitivity and specificity of other possible cutoffs, namely the assay's lower limit of quantitation and the mean +2SD of the seronegative class derived from the mixture modeling [22]. We then used the thresholds derived by the Youden index to assess differences in seroprevalence by age and by site. In addition to deriving a threshold for seropositivity, we estimated seroprevalence directly from individual posterior probabilities of belonging to the higher antibody component, allowing prevalence to vary by age group and study location.

## RESULTS

Among 1497 participants who contributed to the stool carriage survey, 1254 (84%) had a serum sample collected; of these, 319 (25%) came from Kilifi, 451 (36%) from Nairobi, and 484 (39%) from Siaya. Due to limited volumes of sera obtained, we assayed 1252 for O:9 IgG, 1216 for O:4,5 IgG, and 1194 for O:9 and O:4,5 IgA (Supplementary Table 1). One sample had incomplete metadata and was excluded. Participants were broadly comparable across sites with respect to age and sex distribution, although malaria parasitemia and anemia were more prevalent in Siaya (Supplementary Table 2), consistent with known epidemiologic differences among sites. Complete results for all 4 antigen-isotype combinations were available for 1148 participants, who formed the primary analytic dataset.

**Figure 1.**
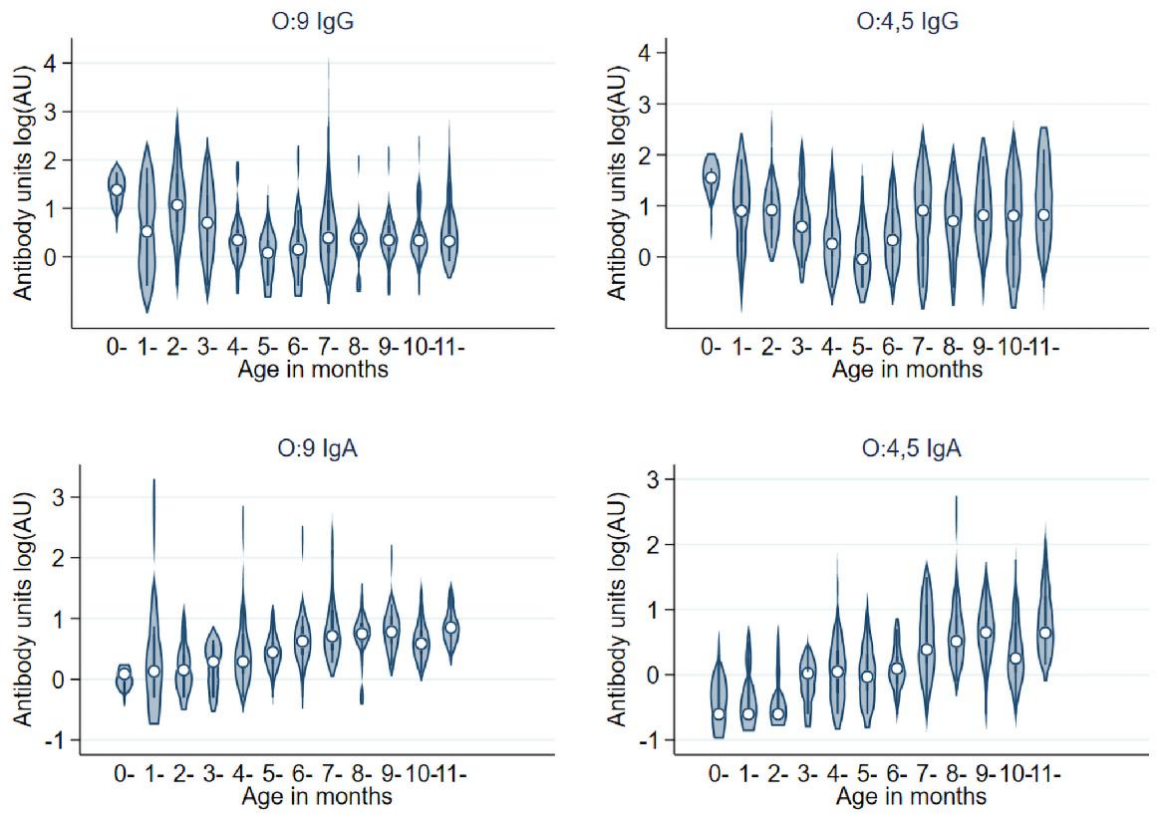
Decay of maternal antibodies (IgG) and early acquisition of antibodies (IgA) among infants. Data are presented as median (circle),Interquartile range (error bars), and distribution (shading). AU, antibody units.

**Figure 2.**
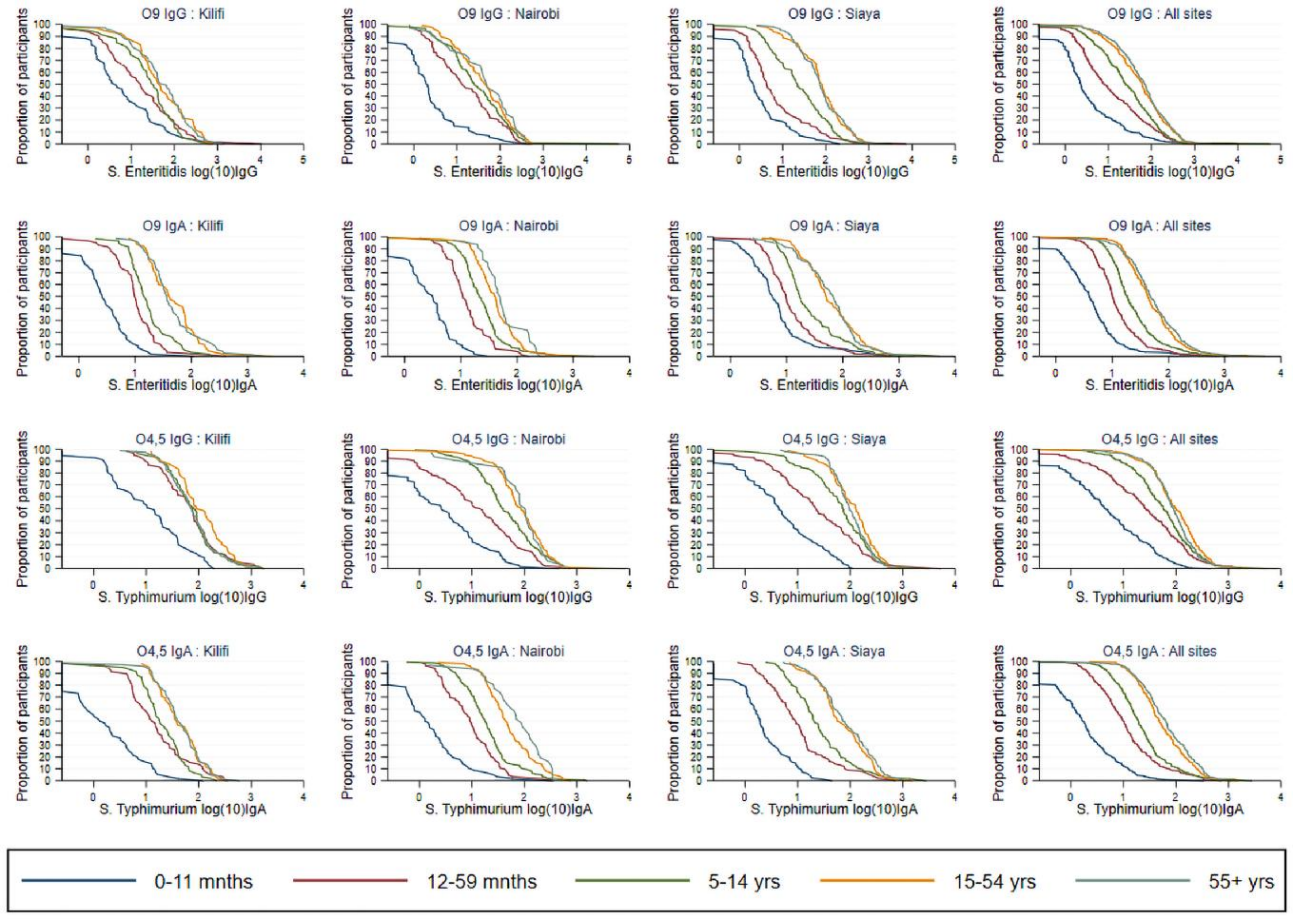
Reverse cumulative distribution curves of the concentrations of O:9 and O:4,5 IgG and IgA by age and site.

Across all sites, age-related patterns differed by isotype. IgG concentrations showed clear evidence of maternally derived antibodies in early infancy, followed by sustained increases with age, whereas IgA concentrations were uniformly low in early life and rose later, consistent with acquisition of endogenous mucosal immune responses following infection (Figure 1). The pooled rate of decay of O:9 and O:4,5 IgG was remarkably similar across locations and serogroups (40% rate per month for the first 6 months of life; Supplementary Figure 3), despite differences in transmission intensity among sites, indicating that early-life differences in antibody concentrations are more likely driven by exposure dynamics after infancy rather than variation in maternal antibody kinetics. Beyond 6 months of age, the IgG and IgA geometric mean concentration increased with age across all sites, as shown by the shift to the right of the reverse cumulative distribution curves with increasing age (Figure 2, Table 1).

**Table 1.** Geometric Mean Concentrations (95% CI) of Antibody Concentrations by Age in Kenya

	Kilifi			Nairobi			Siaya			All Sites		
	No.	GMC	95% CI	No.	GMC	95% CI	No.	GMC	95% CI	No.	GMC	95% CI
O:9 IgG												
0–11 mo	59	6	4–11	73	2	2–4	86	3	2–4	218	3	3–4
12–59 mo	60	17	11–28	96	16	11–23	100	7	5–10	256	12	9–15
5–14 y	59	25	16–38	145	28	21–37	108	23	18–32	312	26	21–31
15–54 y	64	49	32–72	104	41	31–54	82	76	57–101	250	52	44–63
≥55 y	75	51	35–72	32	44	24–80	108	76	60–97	215	61	50–74
O:4,5 IgG												
0–11 mo	55	10	6–16	72	3	2–4	77	5	3–7	204	5	4–6
12–59 mo	60	66	46–93	92	12	8–18	98	23	16–35	250	23	18–30
5–14 y	59	76	56–101	145	44	35–56	103	60	45–80	307	54	46–64
15–54 y	64	108	81–143	102	80	63–103	75	108	85–138	241	95	82–111
≥55 y	75	73	56–63	32	75	46–123	106	99	83–118	213	85	74–99
O:9 IgA												
0–11 mo	57	3	2–4	55	3	2–4	82	7	5–9	194	4	3–5
12–59 mo	59	10	8–13	82	13	11–16	99	12	10–15	240	12	10–13
5–14 y	59	17	14–22	138	23	20–28	108	26	20–33	305	23	20–26
15–54 y	63	48	36–62	99	40	33–48	82	60	47–77	244	48	42–55
≥55 y	73	46	36–60	32	56	41–76	105	58	46–74	210	53	46–62
O:4,5 IgA												
0–11 mo	56	2	1–3	56	2	1–3	82	2	2–3	194	2	1–2
12–59 mo	58	15	11–22	83	10	7–12	98	11	8–15	239	11	10–14
5–14 y	59	19	15–26	139	20	16–24	107	27	21–35	305	22	19–25
15–54 y	62	43	34–54	99	52	41–64	82	73	57–94	243	55	48–64
≥55 y	73	42	32–55	31	73	46–116	108	84	67–105	212	65	55–76

Abbreviation: GMC, geometric mean concentration.

The rate of increase of antibody concentrations by age in each site was estimated in a piecewise regression model with 1 inflection point (Supplementary Figures 1 and 2). Mean O:9 IgG increased at a faster rate in Nairobi than in Kilifi and Siaya to reach its maximum concentration by 2.1 years, while mean O:4,5 IgG increased fastest in Kilifi (Table 2). In Kilifi and Siaya, the rate of increase in O:4,5 IgG was faster than O:9 IgG.

**Table 2.** Piecewise Regression Analysis of the Rate of Increase in Antibody Concentrations by Age in Years at Each Site

Location	Intercept at Age = 0	95% CI	Gradient Age < *p*	95% CI	Breakpoint Age, *P*	95% CI
O:9 IgG						
Kilifi	0.9	.7–1.1	0.1	0–.1	13.8	6.9–20.5
Nairobi	0.5	.3–.7	0.7	.4–1	2.1	1.4–2.8
Siaya	0.5	.4–.6	0.1	.1–.1	17.6	14.3–20.8
O:4,5 IgG						
Kilifi	1.1	.9–1.2	0.4	.2–.6	2.0	1.3–2.7
Nairobi	0.6	.5–.8	0.1	.1–.2	8.7	6.9–10.5
Siaya	0.5	.3–.7	0.3	.2–.4	4.5	3.7–5.4

*P* refers to the age at peak antibody concentration. For the second gradient, where age > *p*, the model identified a flat line (−0.0006 < gradient < 0.006) in all instances (not shown).

The results of the stool carriage survey have been published elsewhere [5]; however, for this subset of 1253 participants who contributed a matched serum sample, 35 (3%) were positive for NTS culture, as highlighted in red in Supplementary Figures 1 and 2. There were 6 serogroup D *Salmonella* isolates (4 were *S* Enteritidis) and 10 serogroup B *Salmonella* isolates (1 was *S* Typhimurium). Nineteen isolates could not be fully typed by the antisera available, yet they were neither serogroup B nor serogroup D. Of 35 NTS isolates, 28 (80%) were from Kilifi and 6 (17%) from Siaya. In Nairobi, only 1 stool sample was positive for NTS, and this was of an undefined serogroup. Among children aged <5 years, 3 carried serogroup D while none carried serogroup B *Salmonella*.

Although antibody concentrations tended to be higher among individuals with homologous serogroup carriage (anti-O:9 for serogroup D *Salmonella* or anti-O:4,5 for serogroup B *Salmonella*), particularly for O:4,5 IgG in participants aged ≥5 years, the small number of carriers limited statistical power and precluded formal inference on causality (Supplementary Table 3A and 3B).

For O:9 and O:4,5 IgG, the 2-component mixture models identified a lower and a higher antibody distribution with distinct means and limited overlap (Supplementary Figure 4, Supplementary Table 4), supporting the interpretation of these components as representing populations with relatively low vs high cumulative exposure. The Youden index defined a threshold of 14.1 AUs for O:9 IgG (87% sensitivity and 87% specificity) and 28.2 AUs for O:4,5 IgG (89% sensitivity and 66% specificity; Supplementary Figures 4 and 5, Supplementary Tables 4 and 5).

**Figure 3.**
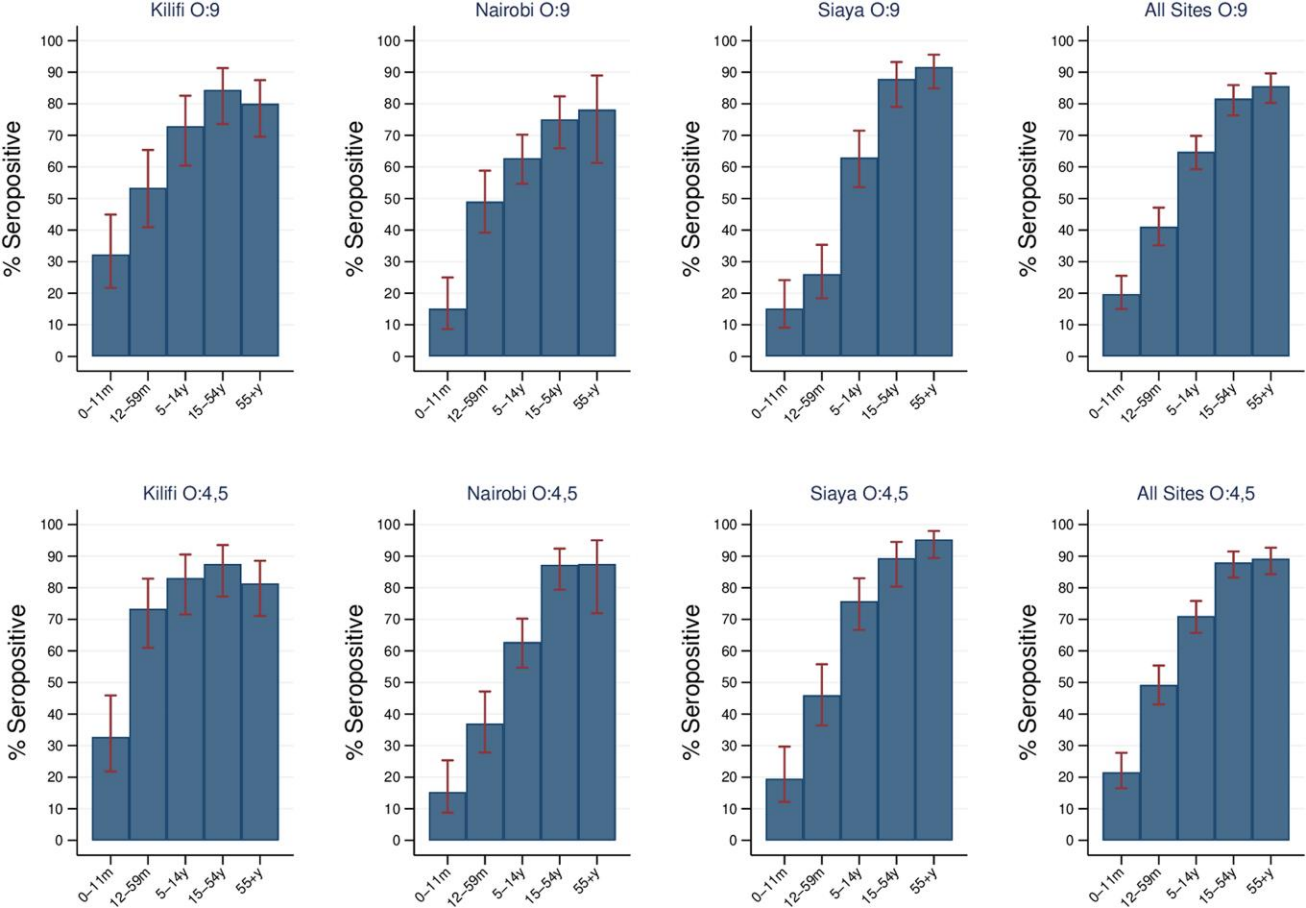
Seroprevalence of O:9 IgG and O:4,5 IgG antibodies by age, using the Youden index as the threshold. Data are presented as percentage (95% CIs).

Like the geometric mean concentrations, seroprevalence increased with age for both antigens (Figure 3). It rose from low levels in infants to higher levels in children aged 5 to 14 years and approached saturation in adults across all locations. By site, the highest seroprevalence was among adults in Siaya for O:9 and O:4,5 IgG. Seroprevalence estimates derived directly from mixture model posterior probabilities, allowing prevalence to vary by age group and location, showed similar age-related and site-specific patterns to those obtained by the cutoff-based approach, including rapid increases in early childhood and convergence to high prevalence in adulthood (Supplementary Figures 5 and 6).

Among 1148 sera tested for O:9 and O:4,5 IgG, 583 (51%) were seropositive for both antigens while 278 (24%) were negative for both antigens (Supplementary Table 6). Patterns of joint seropositivity differed by site. In Kilifi and Siaya, seropositivity to O:9 and O:4,5 IgG was significantly associated, whereas no such association was observed in Nairobi. This suggests that exposure to serogroup B and D *Salmonella* may share common risk factors in rural and high-transmission settings but arise from more distinct transmission pathways in urban Nairobi.

Taken together, these results demonstrate marked age- and site-specific heterogeneity in exposure to NTS, with early loss of maternal protection, rapid acquisition of infection in childhood, and differing transmission dynamics between serogroups and settings.

## DISCUSSION

In this randomly sampled population-level serosurvey of NTS, we have observed that serogroup B (O:4,5) and serogroup D_1_ (O:9) antibodies are present at birth, as maternally derived antibodies, but decline rapidly during early infancy, after which endogenous antibody acquisition begins. This pattern is consistent with observations from other endemic settings [14] and supports a window of heightened vulnerability in the second half of infancy, when maternal protection has waned but immunity from infection has not yet been established.

The lack of IgA responses in early infancy could have several implications. First, it suggests that maternally derived IgG antibodies may have a protective effect against acquisition of infection. Similar observations have been made in cohorts in Vietnam and Malawi, where infections and disease respectively increased in incidence after maternal IgG antibodies declined; these maternal antibodies exhibited in vitro bactericidal activity [14, 25, 26]. Lack of seroconversion in early infancy may also be due to reduced exposure during exclusive breastfeeding. However, in Kilifi, the incidence of invasive NTS disease is highest among neonates [12], especially among those born out of hospital [27], which could be explained by increased mother-to-child transmission of NTS among carrier mothers during vaginal delivery. A maternally administered NTS vaccine could have dual action of reducing mother-to-child transmission of NTS and extending the duration of passive immunity in infants from high-risk areas. In the presence of adequate exposure, a lack of IgA among young infants may also be due to the slow maturation of their immune system [28].

Antibody concentrations and seroprevalence increased with age across all settings, with marked differences in the rate of acquisition among sites. These differences are consistent with known heterogeneity in NTS transmission intensity and suggest that serology captures meaningful variation in cumulative exposure across epidemiologic contexts. For example, the rate of acquisition of O:4,5 IgG was highest in Kilifi, indicating high cumulative incidence of infection with serogroup B *Salmonella*, consistent with known epidemiology of NTS in Kilifi. For O:9 antibodies, the rate of acquisition with age was highest in Nairobi, reflecting a high incidence of serogroup D *Salmonella* infections, despite low carriage and invasive disease incidence [5, 16]. This suggests that O:9 serology may capture exposure to other serogroup D organisms, including *S* typhi [29, 30], or reflect greater cross-reactivity in urban settings. This highlights an important limitation of O-antigen–based serology and reinforces the need for careful interpretation in areas with overlapping *Salmonella* epidemiology.

The relationship between antibody concentrations and fecal carriage of NTS has not been explored previously. We observed higher O:4,5 IgG concentrations among serogroup B carriers than noncarriers, suggesting sustained stimulation and production of antibodies among prevalent carriers. Whether antibodies protect against acquisition of carriage is not clear, as there is no established threshold of protection against carriage. In addition, the low sensitivity of culture could have led to misclassification. A longitudinal study, starting with a naive population and using more sensitive methodologies (eg, polymerase chain reaction) to define carriage, would be required to describe the direction of the association between antibody concentrations and fecal carriage of NTS, if causal. In addition, measurement of antigen-specific IgA antibodies, either from oral fluid or the more challenging fecal samples, may provide a better measure of mucosal immunity to infection or invasion than IgG and IgA from serum samples [31].

The low specificity of the ELISAs may limit the interpretation of our study. The ELISA was designed to detect the O-antigen, a component of lipopolysaccharide found in multiple gram-negative organisms including NTS. A previous study from Denmark demonstrated cross-reactions with antibodies against other pathogens, such as *Escherichia coli*, *Yersinia enterolitica*, and *Campylobacter* sp, in ELISAs targeting the lipopolysaccharide of NTS [32]. Additionally, for NTS, multiple serotypes exist within 1 O-antigen serogroup, and their responses might not be differentiated easily by O-antigen–based serology. Our assay-based threshold illustrated this lack of specificity, as it defined seropositivity as any value above the lower limit of quantitation. Using this threshold, we observed 100% seroconversion during infancy for both antigens, representing a high background incidence of infection with other gram-negative organisms in the environment, including nonpathogenic salmonellae.

The threshold derived from the Youden index maximized sensitivity and specificity [24]. The utility of the mixture model is contingent on the validity of the underlying assumptions. We assumed that the data contained a mixture of 2 populations (seropositive and seronegative), which is the simplest classification possible. Further subclassifications into individuals recently infected and those with waning antibody could be specified if biologically plausible. However, as this is one of the first studies to examine population distributions of anti-NTS antibodies and because our understanding of the responses and waning rates is rudimentary, we selected a 2-population model as the most parsimonious option. This approach was able to separate the IgG distributions into 2 components each, although there was considerable overlap between them. Yet, for IgA, the models could not separate any underlying components, likely reflecting the biological characteristics of serum IgA responses to enteric pathogens, which are typically lower in magnitude, more heterogeneous, and more transient than IgG, resulting in insufficient separation of latent components.

Although mixture models allow seroprevalence to be estimated directly from posterior probabilities without defining a threshold, we deliberately present cutoff-based seroprevalence estimates in the main analysis. At present, no validated serologic threshold exists for NTS, despite active vaccine development. Defining a provisional, data-driven threshold provides a concrete reference point that can be tested, refined, or challenged in future studies using external correlates such as protection from infection or disease. Importantly, seroprevalence estimates derived from posterior probabilities were highly concordant with those obtained by the cutoff-based approach across age groups and locations, indicating that our key epidemiologic inferences are robust to the method used to summarize seroprevalence.

Without assay standardization, we cannot validate our results through comparisons with other studies. Spatial heterogeneity in our results means that it would be imprudent to generalize the results more widely, say, to the greater African population. However, we have provided data on key contributory parameters, such as the prevalence of *falciparum* malaria, anemia, and malnutrition, which could be used to adjust the results when comparing with other populations.

In summary, serologic profiling reveals rapid loss of maternal protection, followed by early and intense exposure to NTS in Kenyan populations, with substantial heterogeneity among settings. These findings support early-life intervention strategies and provide a framework for the development and interpretation of NTS serology in surveillance and vaccine evaluation. In addition, we have highlighted areas that would benefit from further scientific research, including assay standardization and the thresholds for correlates of protection against infection and invasive disease.

## Supplementary Material

jiag114_Supplementary_Data

## Supplementary Data

Supplementary materials are available at *The Journal of Infectious Diseases* online (http://jid.oxfordjournals.org/). Supplementary materials consist of data provided by the author that are published to benefit the reader. The posted materials are not copyedited. The contents of all supplementary data are the sole responsibility of the authors. Questions or messages regarding errors should be addressed to the author.
