# Supplementary material for: Age-Specific Concentrations and Seroprevalence of Antibodies Against Salmonella Enteritidis (O:9) and Salmonella Typhimurium (O:4,5) Across 3 Sites in Kenya
Source: J Infect Dis. 2026 Mar 4;233(5):e1115–23. doi: 10.1093/infdis/jiag114 (PMC13175609; doi:10.1093/infdis/jiag114)

The age-specific concentrations and seroprevalence of antibodies against *Salmonella* Enteritidis (O:9) and *Salmonella* Typhimurium (O:4,5) across three sites in Kenya.

**Authors:**

Esther M Muthumbi^1,2,3, *^, Sean C Elias^4^, Alfred Mwanzu^1^, Agnes Mutiso^1^, Perpetual Wanjiku^1^, Cecilia Mbae^5^, Godfrey Bigogo^6^, Jennifer R. Verani^7^, Stefan Flasche^2,8^, Samuel Kariuki^5^, Calman A. MacLennan^4,9^, J Anthony G Scott ^1,2^.

Affiliations:

1. Kenya Medical Research Institute – Centre for Geographic Medicine Research, Coast, Kilifi, Kenya
2. Department of Infectious Disease Epidemiology, London School of Hygiene and Tropical Medicine, London, United Kingdom
3. Department of Population Health, Aga Khan University, Nairobi, Nairobi, Kenya
4. Jenner Institute, Nuffield Department of Medicine, University of Oxford, Oxford, United Kingdom
5. Kenya Medical Research Institute – Centre for Microbiology Research, Nairobi, Kenya
6. Kenya Medical Research Institute- Centre for Global Health Research, Kisumu, Kenya
7. U.S. Centers for Disease Control and Prevention, Division of Global Health Protection, Nairobi, Kenya
8. Centre for Global Health at Charite Universitaetsmedizin Berlin, Germany
9. Department of Immunology and Immunotherapy, University of Birmingham, Birmingham, UK

*Corresponding author: esther.muthumbi@aku.edu

**List of Tables and Figures.**

Table 1. Number of samples tested for each antigen-antibody combination.

Table 2: Participant characteristics among the subset of participants with complete results on all four isotype-serotype combinations tested (n=1,148).

Table 3A: Geometric mean concentrations of O:9 IgG and IgA antibodies among those with serogroup D carriage and those without serogroup specific carriage.

Table 3B: Geometric mean concentrations of O:4,5 IgG and IgA antibodies among those with serogroup B carriage and those without serogroup specific carriage.

Table 4: Predicted class means and associated class proportions from mixture modelling(Base model, no adjustments).

Table 5: Sensitivity and Specificity of different cut-offs.

Table 6: Association between seroprevalence of O:9 IgG and seroprevalence of O:4,5 IgG

Figure 1A: Scatterplots of antibody concentration by age in years with LOWESS curves fitted: O:9 IgG and IgA

Figure 1B: Scatterplots of antibody concentration by age in years with LOWESS curves fitted: O:4,5 IgG and IgA

Figure 2A: Scatterplots of antibody concentration by age in years (<10y) with LOWESS curves fitted: O:9 IgG and IgA

Figure 2B: Scatterplots of antibody concentration by age in years (<10y) with LOWESS curves fitted: O:4,5 IgG and IgA

Figure 3: Scatter plot with LOWESS showing crude rate of decay in maternal antibodies by age at each site.

Figure 4: Histograms showing the observed vs predicted densities from a 2-component mixture model

Figure 5: Receiver Operating Characteristic (ROC) Curve of O:9 and O:4,5 IgG concentrations.

Figure 6: Density plots for the fitted two-component mixture model, adjusted for age-group and location.

Figure 7: Seroprevalence by age group and location. Clustered bars show posterior median seroprevalence by age group, stratified by location, with 95% credible intervals.

Table 1. Number of samples tested for each antigen-antibody combination.

|  | O:9 IgG | O:4,5 IgG | O:9 IgA | O:4,5 IgA |
| --- | --- | --- | --- | --- |
| Vials retrieved | 1254 | 1254 | 1254 | 1254 |
| Insufficient Volume | 1 | 2 | 53 | 55 |
| Failed CV criterion (>20) | 1 | 12 | 7 | 5 |
| Plate Failed* | - | 22 | - | - |
|  | 1252 | 1216 | 1194 | 1194 |

*(R^2^<0.994 or Controls out of range)

Table 2: Participant characteristics among the subset of participants with complete results on all four isotype-serotype combinations tested (n=1,148).

|  | Kilifi | | Nairobi | | Siaya | | Total | |
| --- | --- | --- | --- | --- | --- | --- | --- | --- |
|  | n | % | n | % | n | % | n | % |
| N | 302 |  | 397 |  | 449 |  | 1,148 |  |
| Age |  |  |  |  |  |  |  |  |
| 0-11m | 50 | 17 | 54 | 14 | 73 | 16 | 177 | 15 |
| 12-59m | 58 | 19 | 78 | 20 | 96 | 21 | 232 | 20 |
| 5-14y | 59 | 20 | 137 | 35 | 102 | 23 | 298 | 26 |
| 15-54y | 62 | 21 | 97 | 24 | 75 | 17 | 234 | 20 |
| >55y | 73 | 24 | 31 | 8 | 103 | 23 | 207 | 18 |
|  |  |  |  |  |  |  |  |  |
| Sex, male | 123 | 41 | 198 | 50 | 197 | 44 | 518 | 45 |
| MUAC <11.5cm in under 5y† | 1 | 0.9 | 22 | 17 | 5 | 3 | 28 | 7 |
| Number tested for Hb concentration* | 291 | 96 | 347 | 87 | 213 | 47 | 851 | 74 |
| Anaemia (Hb<10g/dl)^*^ | 57 | 20 | 17 | 5 | 19 | 9 | 93 | 11 |
| Number tested for *P. falciparum* malaria* | 295 | 98 | 357 | 90 | 441 | 98 | 1,093 | 95 |
| *P. falciparum* positive by HRP2^*^ | 21 | 7 | 9 | 3 | 122 | 28 | 152 | 14 |

MUAC – Mid Upper Arm Circumference

† Only measured for children under 5years of age

*Number tested for Hb or HRP-2 as a subset of the total participants

The median age (and interquartile range) for the participants were 11y (3-54) in Kilifi, 9y (3-23) in Nairobi, 10y (3-48) in Siaya and 9.7y (2.8-34) for all sites combined

Table 3A: Geometric mean concentrations of O:9 IgG and IgA antibodies among those with serogroup D carriage and those without serogroup specific carriage.

|  |  | Carriers of serogroup D | |  | Carriers of other serogroups | |  | No carriage | |  | GMC Ratio | |
| --- | --- | --- | --- | --- | --- | --- | --- | --- | --- | --- | --- | --- |
| Antibody | Age | n | GMC (95% CI) |  | n | GMC(95% CI) |  | n | GMC(95% CI) |  | Serogroup D vs other serogroup | Serogroup D vs No carriage |
| O:9 IgG | <5 years | 3 | 27 (0, 4324) |  | 5 | 8 (1-52) |  | 466 | 6 (5-8) |  | 3.3 (0-747) | 4.2 (0.5-37.1) |
|  | 5+ years | 3 | 55(2.4, 1277) |  | 25 | 53 (25-116) |  | 749 | 41 (36-46) |  | 1.0 (0.1-10.3) | 1.4 (0.2-8.1) |
| O:9 IgA | <5 years | 2 | 27 (0-1500) |  | 5 | 8 (3-19) |  | 427 | 7 (6-8) |  | 3.6 (0.3-37.8) | 3.8 (0.7-22.5) |
|  | 5+ years | 3 | 38 (8-190) |  | 25 | 34 (23-51) |  | 731 | 37 (34-40) |  | 1.1 (0.3-3.8) | 1.0 (0.3-3.9) |

Table 3B: Geometric mean concentrations of O:4,5 IgG and IgA antibodies among those with serogroup B carriage and those without serogroup specific carriage.

|  |  | Carriers of serogroup B | |  | Carriers of other serogroups | |  | No carriage | |  | GMC Ratio | |
| --- | --- | --- | --- | --- | --- | --- | --- | --- | --- | --- | --- | --- |
| Antibody | Age | n | GMC |  | n | GMC |  | n | GMC |  | Serogroup B vs other serogroup | Serogroup B vs No carriage |
| O:4,5 IgG | <5 years | - | - |  | 7 | 14 (2-88) |  | 447 | 11 (9-14) |  | - | - |
|  | 5+ years | 10 | 177 (74-424) |  | 18 | 88 (47-163) |  | 733 | 72 (66-79) |  | 2.0 (0.7-5.5) | 2.4 (1.1-5.4) |
| O:4,5 IgA | <5 years | - | - |  | 7 | 6 (2-16) |  | 426 | 5 (4-6) |  | - | - |
|  | 5+ years | 10 | 59 (16-212) |  | 18 | 27 (12-61) |  | 732 | 40 (36-44) |  | 2.2 (0.5-8.7) | 1.5 (0.7-3.3) |

Figure 1A: Scatterplots of antibody concentration by age in years with LOWESS curves fitted: O:9 IgG and IgA


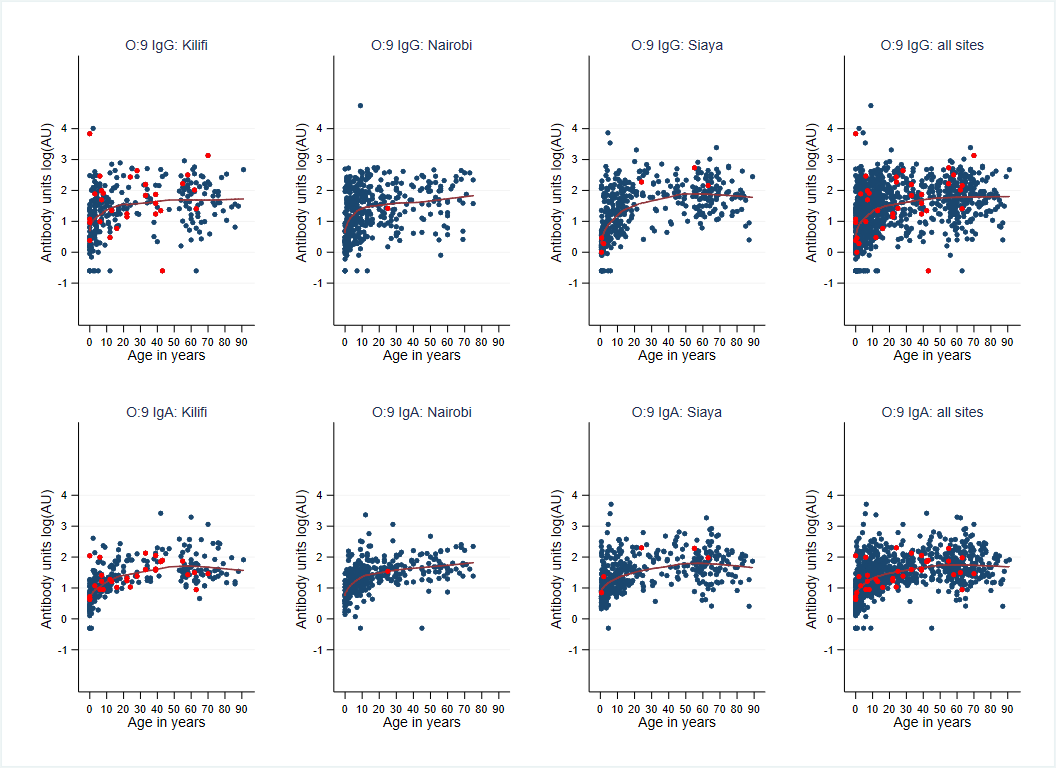


Red dots represent participants with positive stool carriage of NTS;

Figure 1B: Scatterplots of antibody concentration by age in years with LOWESS curves fitted: O:4,5 IgG and IgA


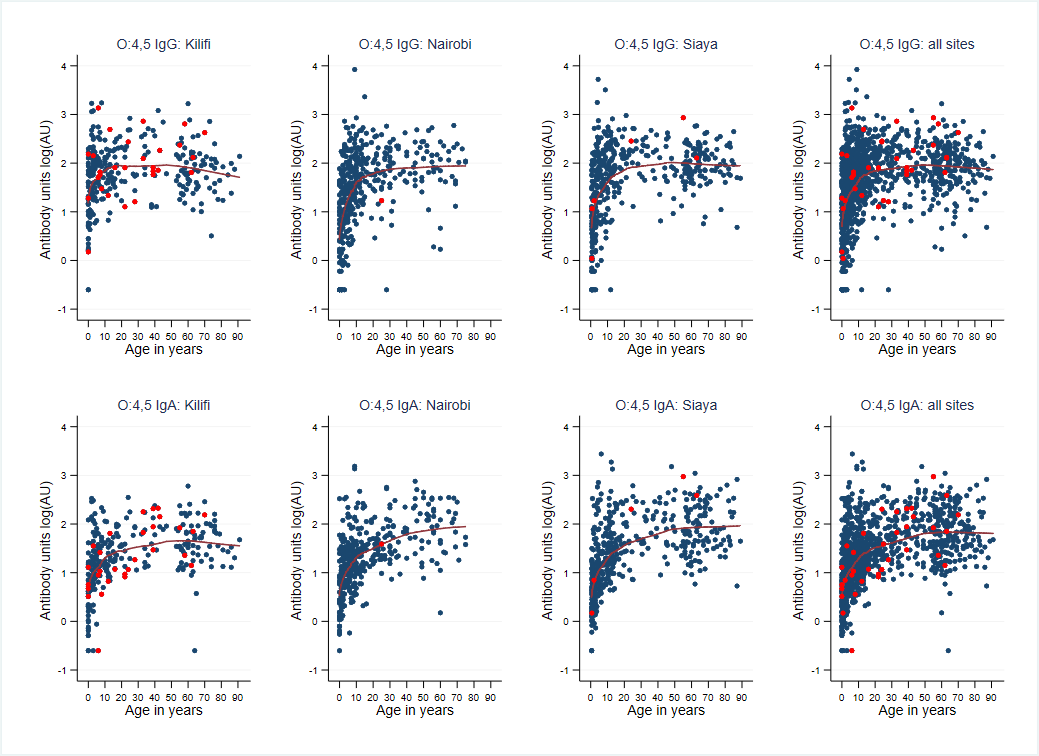


Red dots represent participants with positive stool carriage of NTS

Figure 2A: Scatterplots of antibody concentration by age in years (<10y) with LOWESS curves fitted: O:9 IgG and IgA


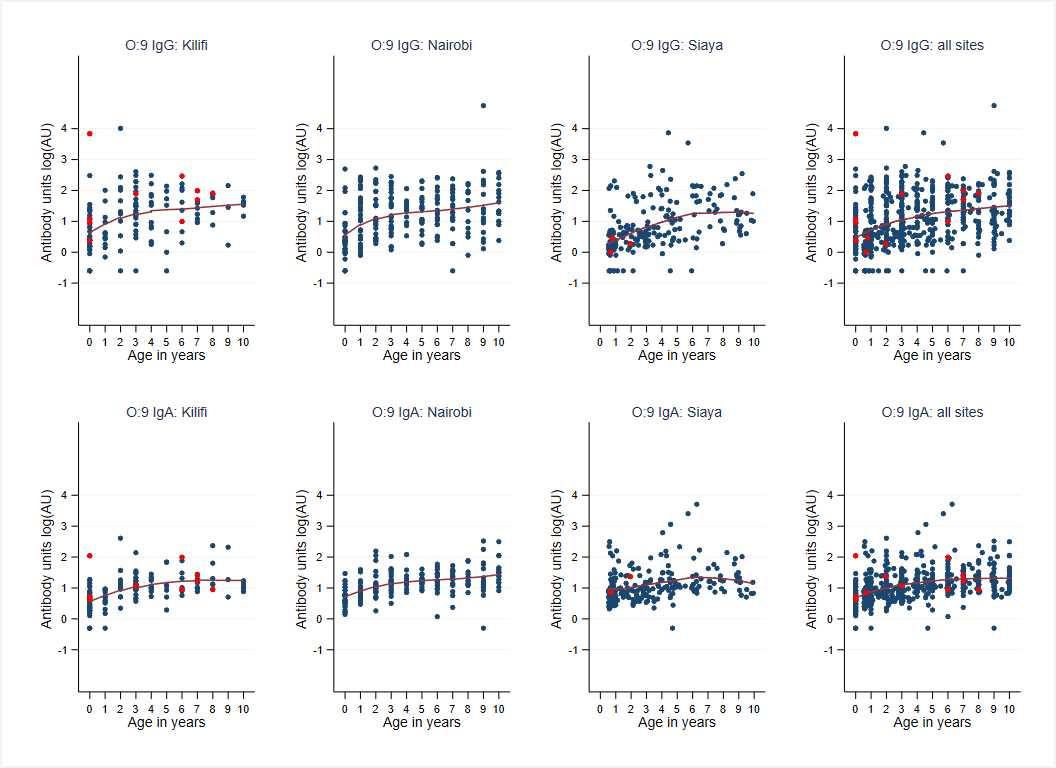


Red dots represent participants with positive stool carriage of NTS

Figure 2B: Scatterplots of antibody concentration by age in years (<10y) with LOWESS curves fitted: O:4,5 IgG and IgA


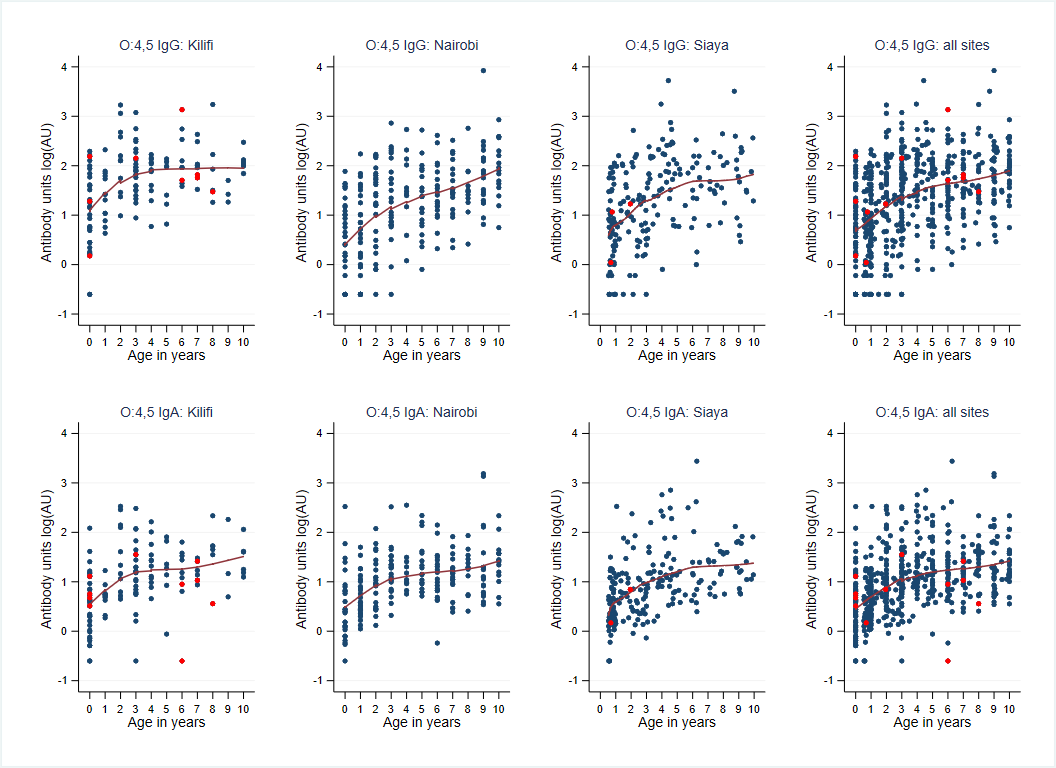


Red dots represent participants with positive stool carriage of NTS

Figure 3: Scatter plot with LOWESS showing crude rate of decay in maternal antibodies by age at each site.

The numbers represent the crude rate of decay (and 95% CI) estimated for each site and serotype by linear regression.

Figure 4: Histograms showing the observed vs predicted densities from a 2-component mixture model


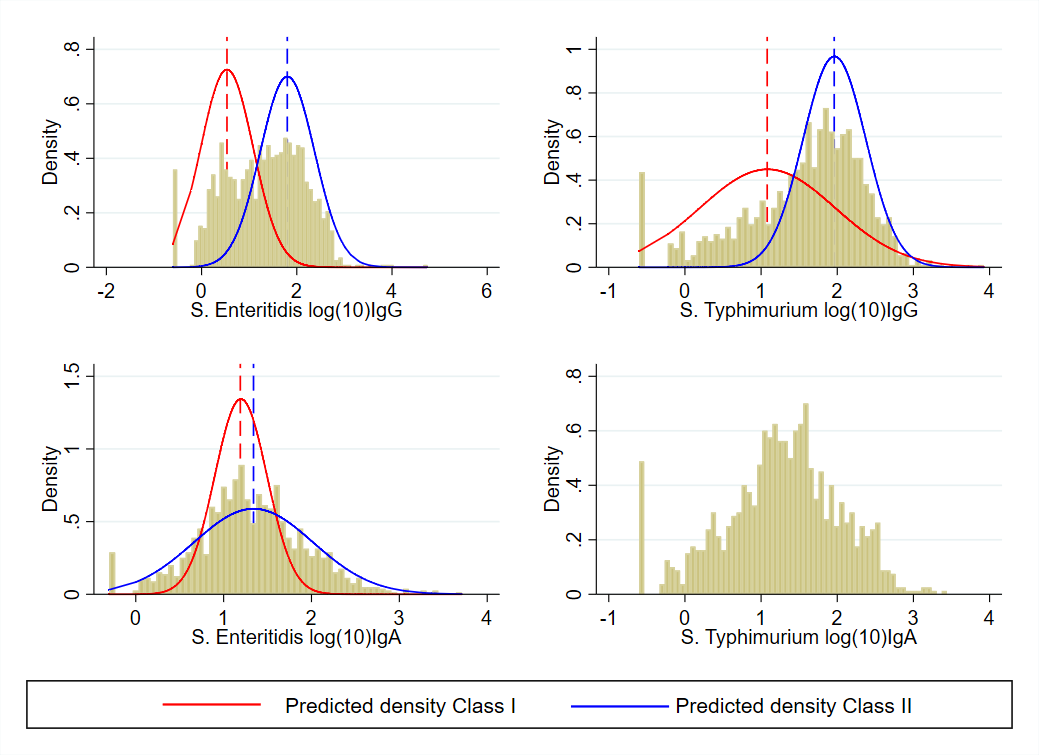


Table 4: Predicted class means and associated class proportions from mixture modelling (Base model, no adjustments).

|  | O:9 IgG | |  | O:4,5 IgG | |  | O:9 IgA | |
| --- | --- | --- | --- | --- | --- | --- | --- | --- |
|  | Class 1 | Class 2 |  | Class 1 | Class 2 |  | Class 1 | Class 2 |
| Predicted Mean (Log AU)  (SD) | 0.53  (0.5) | 1.8  (0.6) |  | 1.08  (0.9) | 1.96  (0.4) |  | 1.19  (0.3) | 1.34  (0.7) |
| Predicted Proportion  (95% CI) | 0.38  (0.27-0.51) | 0.61  (0.48-0.72) |  | 0.45  (0.36-0.54) | 0.55  (0.45-0.64) |  | 0.22  (0.08-0.44) | 0.78  (0.56-0.92) |

Figure 5: Receiver Operating Characteristic (ROC) Curve of O:9 and O:4,5 IgG concentrations.


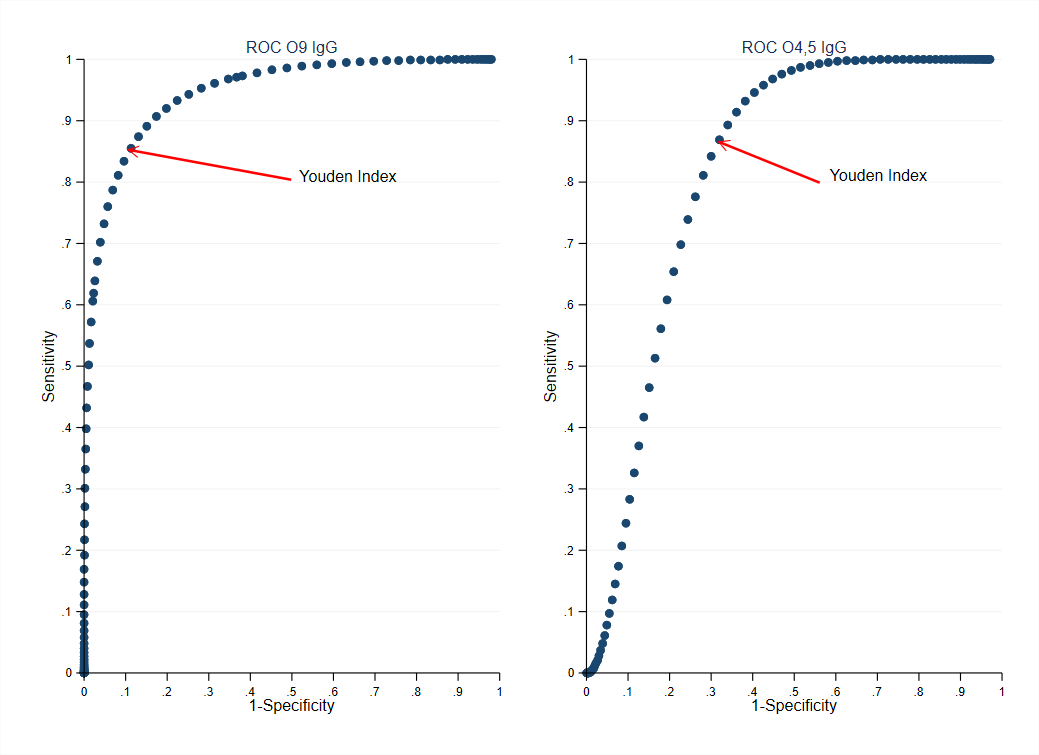


Table 5: Sensitivity and Specificity of different cut-offs.

|  | O:9 IgG | | |  | O:4,5 IgG | | |
| --- | --- | --- | --- | --- | --- | --- | --- |
|  | Mean of Class 1 + 2SD | Youden Index | LLOQ |  | Mean of Class 1 + 2SD | Youden Index | LLOQ |
| *Thresholds* |  |  |  |  |  |  |  |
| Cut off LogAU | 1.63 | 1.15 | -0.3 |  | 2.88 | 1.45 | -0.3 |
| Cut off AU | 42.7 | 14.1 | 0.5 |  | 758.6 | 28.2 | 0.5 |
| *Test statistics* |  |  |  |  |  |  |  |
| Sensitivity | 62% | 87% | 99% |  | 1% | 89% | 99% |
| Specificity | 97% | 87% | 48% |  | 98% | 66% | 7% |
| *Seroprevalence* |  |  |  |  |  |  |  |
| 0-11 m | 21/218 (10%) | 43/218 (20%) | 191/218 (87%) |  | 0/204 (0%) | 44/204 (22%) | 176/204 (86%) |
| 12-59 m | 65/256 (25%) | 105/256 (41%) | 250/256 (98%) |  | 5/250 (2%) | 123/250 (49%) | 240/250 (96%) |
| 5-14 y | 125/312 (40%) | 202/312 (65%) | 307/312 (98%) |  | 6/307 (2%) | 218/307 (71%) | 306/307 (100%) |
| 15-54 y | 146/250 (58%) | 204/250 (82%) | 249/250 (100%) |  | 4/241 (2%) | 212/241 (88%) | 240/241 (100%) |
| 55+ y | 133/215 (62%) | 184/215 (86%) | 214/215 (100%) |  | 3/213 (1%) | 190/213 (89%) | 213/213 (100%) |

Table 6: Association between seroprevalence of O:9 IgG and seroprevalence of O:4,5 IgG

|  | Kilifi | Nairobi | Siaya | All |
| --- | --- | --- | --- | --- |
| N (number of paired samples) | 302 | 397 | 449 | 1148 |
| Seropositive for both antibodies | 170 (56%) | 175 (44%) | 238 (53%) | 583 (51%) |
| Seronegative for both antibodies | 48 (16%) | 104 (26%) | 126 (28%) | 278 (24%) |
| O:4,5 seropositive, O:9 seronegative | 52 (17%) | 63 (16%) | 63 (14%) | 178 (16%) |
| O:9 seropositive, O:4,5 seronegative | 32 (11%) | 55 (14%) | 22 (5%) | 109 (9%) |
| McNemar Chi^2^ | 4.76 | 0.54 | 19.78 | 16.6 |
| p-value | 0.03 | 0.461 | <0.001 | <0.001 |

seroprevalence differed significantly in Siaya and Kilifi but not in Nairobi, meaning the risk factors for 0:9 and 0:4,5 infections are not shared in Nairobi, while in Kilifi and Siaya the two serogroups have shared risk factors.

As additional sensitivity analyses, we analysed IgG responses to the Salmonella O:9 and O:4,5 antigens using a two-component mixture modelling approach as earlier but this time allowing seroprevalence to vary by age-group and location. Models were fitted in a Bayesian framework using the **brms** R package with Stan’s Hamiltonian Monte Carlo sampler. Seroprevalence was defined as the posterior probability of belonging to the higher-IgG component, summarised using posterior medians and 95% credible intervals. Figure 6 and 7 represent model outputs from this analysis.

Figure 6. Density plots for the fitted two-component mixture model, adjusted for age-group and location

Histogram and kernel density of observed log(IgG) (black solid line) overlaid with the fitted mixture density (red solid line) and the two component densities, low (green solid line) and high (blue solid line). Vertical dotted lines indicate component means, demonstrating clear separation of the lower- and higher-IgG components.

A. O:9 IgG


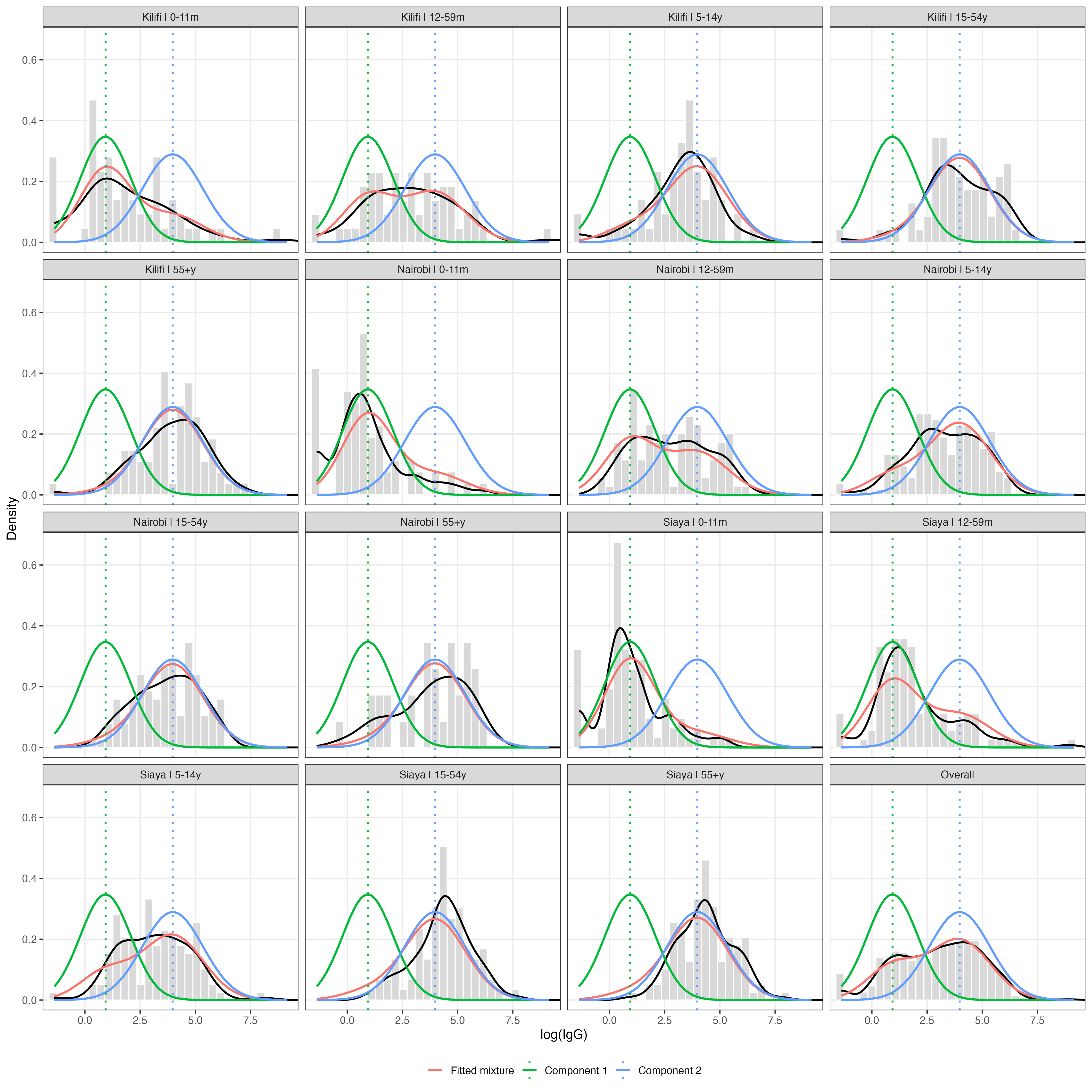


B: O:4,5 IgG


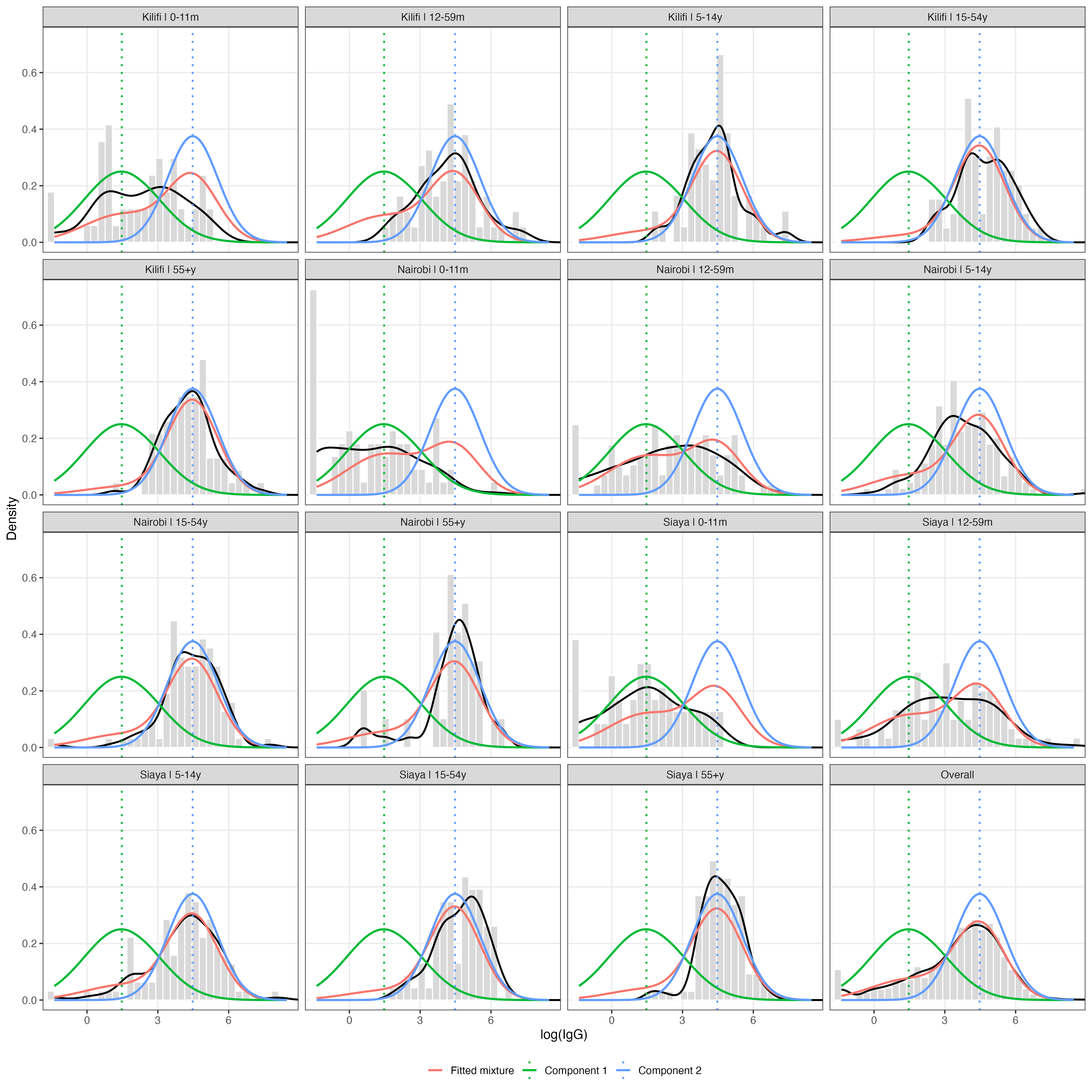


Figure 7: Seroprevalence by age group and location. Clustered bars show posterior median seroprevalence by age group, stratified by location, with 95% credible intervals.

A: O:9 IgG


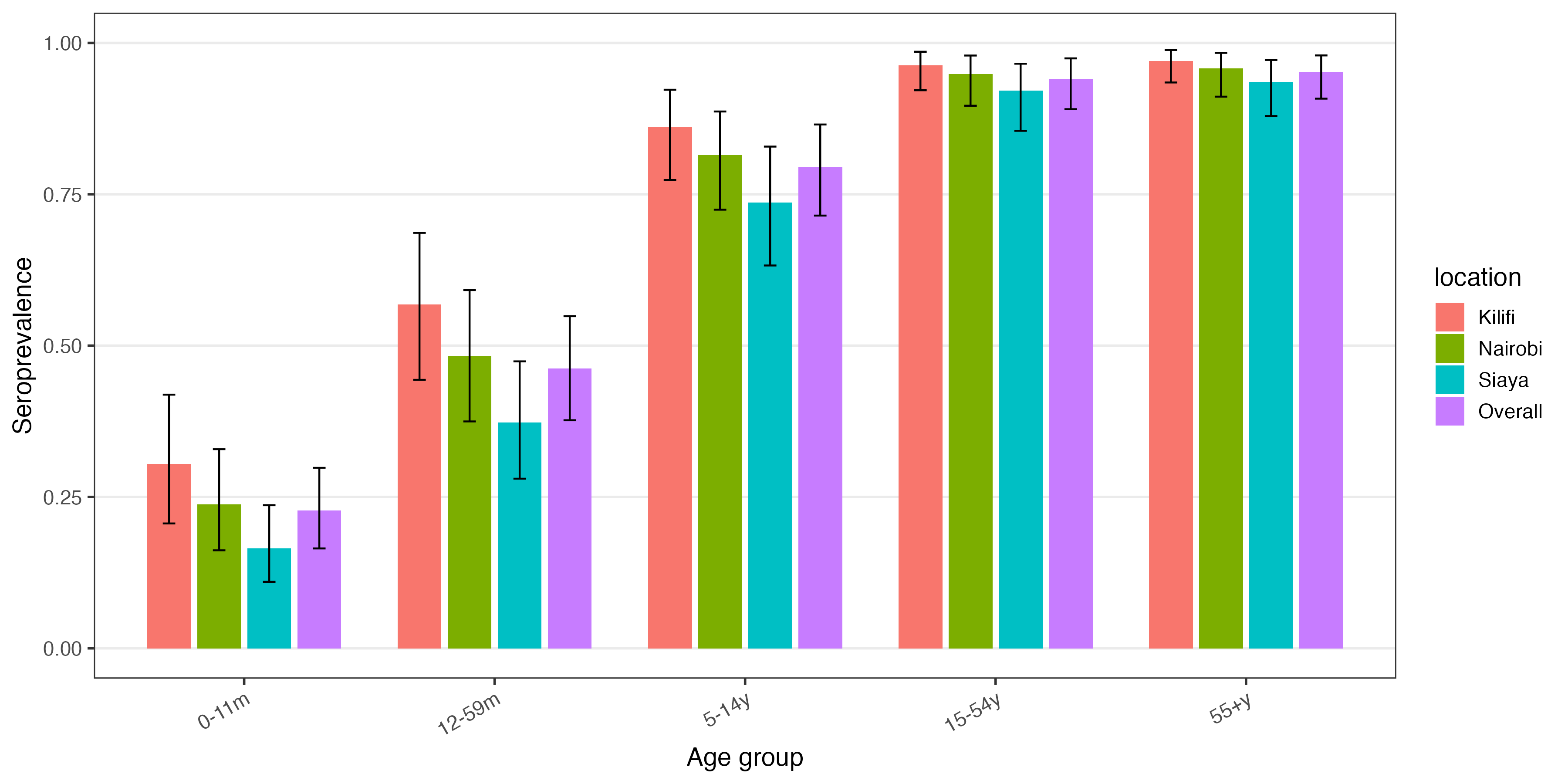


B: O:4,5 IgG


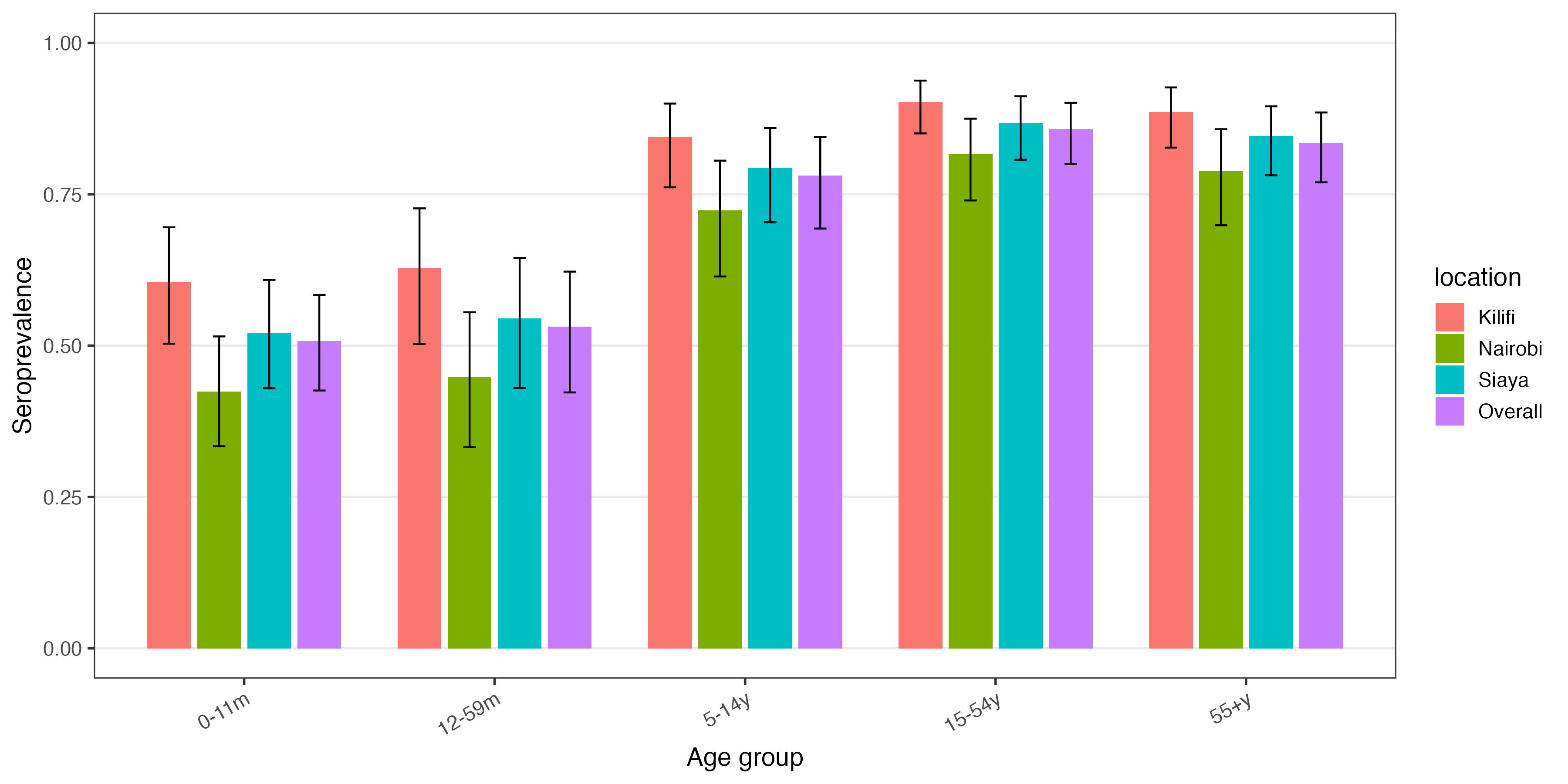

Supplement: jiag114_Supplementary_Data [file jiag114_supplementary_data.docx]
